# Polymorphisms of interleukin-21 and interleukin-21-receptor genes confer risk for autoimmune thyroid diseases

**DOI:** 10.1186/1472-6823-13-26

**Published:** 2013-07-29

**Authors:** Jian Zhang, Wan Xia Xiao, Yuan Feng Zhu, Fatuma Said Muhali, Ling Xiao, Wen Juan Jiang, Xiao Hong Shi, Lian Hua Zhou, Jin An Zhang

**Affiliations:** 1 Department of Clinical Laboratory, Jinshan Hospital of Fudan University, Shanghai 201508, China; 2 Internal Medicine Department, Xi’an Aviation Group Hospital, Xi’an 710021, China; 3 Endocrinology Department, Jinshan Hospital, Fudan University, 1508 Longhang Road, Shanghai 201508, China; 4 Endocrinology Department, Weinan Central Hospital, Weinan, Shaanxi 714000, China

**Keywords:** Interleukin-21 (IL-21), Interleukin-21 receptor (IL-21R), Graves’ disease, Hashimoto’s thyroiditis, Single nucleotide polymorphism (SNP)

## Abstract

**Background:**

The abnormality of interleukin-21 (IL-21)-IL-21-receptor (IL-21R) system has been found in many autoimmune diseases including autoimmune thyroid diseases (AITDs). In this study, we investigated whether polymorphisms of the IL-21 and IL-21R are associated with Graves’ disease (GD) and Hashimoto’s thyroiditis (HT), two major forms of AITDs, among a Chinese population.

**Methods:**

Rs907715, rs4833837, rs2221903 and rs2055979 of the IL-21 gene and rs3093301 and rs2285452 of the IL-21R gene were explored in a case–control study including 405 GD, 228 HT patients and 242 controls. These genes were genotyped by the PCR and restriction fragment length polymorphism (RFLP) analysis and the MASS spectrometry method.

**Results:**

For IL-21 gene, we identified and confirmed a higher prevalence of A alleles of rs2221903 (P = 0.018, OR = 1.50 95% CI = 1.07-2.09) in GD patients. We also found a significant association between rs2221903 and HT (allele: P = 0.009, OR = 1.69 95% CI = 1.13-2.51; genotype: recessive P = 0.021, OR = 11.72 95% CI = 1.46-94.13). For the IL-21R gene, compared with controls, the genotype frequencies of rs3093301 and rs2285452 were significantly different in HT patients using dominant genetic model (P = 0.023, OR = 1.61 95% CI = 1.07-2.42; P = 0.031, OR = 1.71 95% CI = 1.05-2.80, respectively). Furthermore, the haplotype AA containing the major alleles of rs4833837 and rs2221903 was associated with increased susceptibility to GD with an OR of 1.50(95% CI =1.08-2.09, P = 0.016), and to HT with an OR of 1.69(95% CI =1.14-2.52, P = 0.009).

**Conclusion:**

Our results indicated that the SNPs of the IL-21 gene is associated with the development of GD. In addition, we found that individuals with the SNPs of the common IL-21 and IL-21R may have higher risk of HT.

## Background

Autoimmune thyroid diseases (AITDs), the thyroid-specific autoimmune disorders, affect about 5% of the population. The etiology of AITDs includes genetic and environmental factors resulting in immune abnormality and the development of AITDs.

In 2000, the interleukin-21(IL-21)-IL21-receptor (IL-21R) system was discovered [1]. IL-21 and IL-21R are located on human chromosome 4q26-27 and 16p11, respectively. The IL-21, preferentially produced by CD4 + T cells, has various functions, such as driving B cells to differentiate into memory cells and ultimately plasma cells, augmenting T cells’ proliferation, and promoting the activity of natural killer (NK) cells [2]. In accordance with these roles of IL-21, IL-21R is mainly expressed in B cells, T cells and NK cells, keratinocytes, and some of myeloid cells. Animal and clinical studies demonstrated the dysregulations of IL-21 and IL-21R in autoimmune diseases. For example, compared with IL-21R-competent BXSB-Yaa mice for multiple parameters of SLE, the IL-21R-deficient Yaa mice showed none of the abnormalities characteristic of systemic lupus erythematosus (SLE) [3]. IL-21R is overexpressed in the inflamed synovial membrane and in peripheral blood or synovial fluid leukocytes of rheumatoid arthritis (RA) patients [4]. And the evident increase of serum IL-21 level in primary Sjogren’s syndrome (pSS) patients has positive correlation with the levels of gamma-globulin and erythrocyte sedimentation rate [5].These suggests that IL-21 and IL-21R may play a critical role in the pathogenesis of autoimmune diseases. Recently, H.Y. Jia [6] also reported that an increase of serum IL-21 level in patients with primary GD.

In recent years, the associations between the IL-21 gene or IL-21R gene polymorphisms and autoimmune diseases have been gradually reported. In a HapMap-CEU population, a large block (480 kb) of linkage disequilibrium encompassing KIAA1109/Tenr/IL2/IL21 showed genetic associations with type 1 diabetes mellitus (T1DM) [7,8], RA [8], juvenile idiopathic arthritis (JIA) [9], psoriasis and psoriatic arthritis(PA) [10]. Similarly, the polymorphisms of the IL2/IL21 gene region likely to confer to the susceptibility to coeliac disease in Scandinavia families [11] and a US population [12], to ulcerative colitis in a North American and an Italian cohort [13], and to multiple sclerosis in a Spanish population [14]. After the SNPs in the IL-21 gene were analyzed, the significant differences of rs907715 and rs2221903 allele and genotype frequencies were found between SLE and controls [15]. In addition to these reports, an association between microsatellite polymorphisms of the IL-21R and diabetes in Japanese patients, between rs3093301 and rs2285452 in the IL-21R gene region and SLE in the European-derived cohort were documented [16].

Therefore, it is reasonable to speculate that IL-21 and IL-21R are the candidate genes for AITDs. Plagnol [17] reported that the rs2069763 in chromosome 4q27 were associated with the GD susceptibility in a Caucasian cohort. And a recent study that indicated that rs907715 of IL-21 gene was associated with GD in a Chinese population [6].

In this study, additional variants of the IL-21 gene and two SNPs of the IL-21R gene in addition to previously reported variants were investigated in two subtypes of AITDs including GD and HT.

## Methods

### Patients and controls

AITDs patients and healthy controls were enrolled from the First Affiliated Hospital, Medical School of Xi’an Jiaotong University. A total of 633 independent patients with AITDs, including 405 GD and 228 HT, and 242 unrelated healthy controls were recruited. GD and HT were diagnosed based on clinical and laboratory evidence of hyperthyroidism and hypothyroidism respectively and diffuse goitre, supported by the presence of antithyroglobulin antibody (TgAb) and/or antithyroid peroxidase antibody (TPOAb) and/or exophthalmos. The controls were healthy subjects without clinical evidence or family history of any AITDs. This study was approved by the ethics committee of Jinshan hospital of Fudan University. All patients and control subjects were asked to sign an informed consent.

### Genotyping

Genomic DNA was extracted from peripheral blood leukocytes using the Nucleon Bacc kit from TianGen Biotech CO. LTD (Beijing, China).

Rs907715, rs4833837, rs2221903 and rs2055979 spanning a large region which captured all common variation in the IL-21 gene and rs3093301 and rs2285452 which were only two tag-SNPs of IL-21R gene confirmed to be associated with SLE in European-derived cohort were selected from the published SNP database (http://www.ncbi.nlm.nih.gov/SNP). All of these SNPs were validated polymorphisms, and have a minor allele frequency(MAF) of more than 1%. Rs907715, rs4833837, and rs222190 are located on intron 2 of IL-21R and rs2055979 on intron 3. For IL-21R gene, rs3093301 is in intron 2 and rs2285452 in exon 10.

All of the four IL-21 SNPs and rs2285452 were typed using mass spectrometry method (Shanghai Benegene Biotechnologies CO. Ltd. Shanghai, China). Rs3093301 was typed using PCR-restriction fragment length polymorphism (PCR-RELP) method. The appropriate fragment of rs3093301 in the IL-21R gene was amplified using specific primers (forward: 5′-AATTGCTCCT CAGCAGATCC-3′, reverse: 5′-GCATCAGCCTCCCGAGTAG-3′). The PCR reaction was performed in a 25μl mixture, containing 100ng genomic DNA and 0.1 mM solution of each primer, 12.5 μl 2*Taq PCR MasterMix (TianGen Biotech CO. LTD, Beijing, China) and 9.5 μl ddH_2_O. PCR was carried out with initial denaturation for 5 min at 95°C, followed by 37 cycles of annealing for 30 seconds at 60°C, extension for 30 seconds at 72°C and denaturation for 30 seconds, and a final extension for 5 min at 72°C. The product (383 bp) was digested with restriction enzyme NIaIII (Fermentas Life Sciences) at 37 centigrade for 10 hours, and analyzed on a 3% agarose gel. There were three profiles of the digested fragments: 210 bp, 44 bp, and 129 bp indicating the presence of TT, two fragments of 254 bp and 129 bp indicating the presence of CC, and four fragments of 254 bp, 210 bp, 129 bp and 44 bp representing TC genotype.

### Statistical analysis

In this study, we used a population-based case–control method. Statistical analysis was performed using Haploview 4.1 and SPSS 11.0. Hardy-Weinberg equilibrium (HWE) of each SNP was analyzed in controls using Goodness-of-fit test. Categorical data were analyzed by chi-square or Fisher’s exact test. For continuous data, Student’s t-test was used for the normalized data, and non-parametric test was used for non-normalized data. Furthermore, corrected P value and gene-gene interaction were analyzed using logistic regression analysis. In this study, a two-tailed P value less than 0.05 was considered to be significantly difference.

## Results

Four SNPs (rs907715, rs4833837, rs2221903 and rs2055979) of the IL-21 gene and two SNPs (rs3093301 and rs2285452) of the IL-21R gene were typed in 405 GD and 228 HT patients and 242 controls. For the tested SNPs, genotyping successful rate was more than 96.9%. All of the 6 genotyped SNPs showed a MAF more than 7% and HWE analysis in the three groups showed P values of more than 0.05 (seen in Table 1). When comparing age and sex distributions between patients and controls, we found the control group matched well with the GD group, but not with HT group (seen in Table 2). The distributions of the SNPs genotypes in HT patients were analyzed using logistic regression analysis adjusted for sex and age when compared with the controls.

**Table 1:** Allele distributions and HWE P value of the four SNPs in IL21 gene and two SNPs in IL-21R gene

**SNP ID**	**HWE P value**			**Associated allele**	**Count (frequency %)**			**GD P value (OR,95% CI)**	**HT P value (OR,95% CI)**
	**Control**	**GD**	**HT**		**Control**	**GD**	**HT**		
IL-21									
rs907715	0.634	0.968	0.085	A	208(44.6)	348(44.5)	210(46.9)	0.963	0.497
rs4833837	0.999	0.300	0.715	G	56(11.6)	86(10.6)	44(9.6)	0.606	0.340
rs2221903	0.055	0.329	0.715	A	405(84.7)	721(89.2)	412(90.4)	0.018 (1.50, 1.07-2.09)	0.009 (1.69,1.13-2.51)
rs2055979	0.507	0.999	0.484	T	186(38.9)	312(39.7)	160(36.5)	0.782	0.458
IL-21R									
rs3093301	0.427	0.732	0.172	T	228(47.1)	387(48.0)	192(42.3)	0.752	0.138
rs2285452	0.326	0.510	0.830	A	55(11.3)	89(11.0)	36(7.9)	0.797	0.068

**Table 2:** Age and sex characteristics of GD and HT patients and controls

**Group**	**Age**			**Sex**		
	**Kolmogorov - Smirnov P value**	**Z score**	**P value**	**Male/female counts**	**χ2**	**P value**
control	<0.001	--	--	67/175	--	--
GD	<0.001	-0.487	0.626	114/291	0.016	0.899
HT	0.200	-2.599	0.009	27/201	18.410	1.7E-5

### Association of the IL-21 gene polymorphisms with GD and HT

A genetic association between GD and HT and one of the four genotyped IL-21 SNPs was found in this study. For the SNP rs2221903 located in the intron 2 regions, the frequency of A allele was 89.2% in GD and 90.4% in HT, respectively, significantly higher than 84.7% in healthy controls (GD: P = 0.018, OR = 1.495 95% CI = 1.070-2.086; HT: P = 0.009, OR = 1.69 95% CI = 1.13-2.51). In contrast to these results, the allele frequencies of rs907715, rs4833837 and rs2055979 were similar between patients and controls (Table 1). As the distributions of rs907715, rs4833837, rs2221903 and rs2055979 genotypes in patients and controls are shown in Table 3. For rs2221903, the frequencies of the genotypes carrying risk allele (A) were higher both in GD (AA: 80.2%, GA: 18.1%) and HT (AA: 81.1%, GA:18.4%) patients than in controls (AA: 73.6%, GA: 22.2%), but statistical significance was only found when the recessive genetic model was used in HT patients (AA + GA vs. GG, P = 0.021, OR = 11.72 95% CI = 1.46-94.13), in comparison with the controls. In this study, no significant association was observed between GD or HT and rs907715, rs4833837 or rs2055979 genotypes while different genetic models were applied for analysis.

**Table 3:** Genotype distributions of the four SNPs of IL21 gene and two SNPs of IL-21R gene

**SNP ID**	**Geno-type**	**Control**	**GD**				**HT**			
		**Count (frequency%)**	**Count (frequency %)**	**Additive P value**	**Dominant P value**	**Recessive P value**	**Count (frequency %)**	**Additive P value**	**Dominant P value (OR,95% CI)**	**Recessive P value (OR,95% CI)**
IL-21										
rs907715	GG	69(29.6)	121(30.9)	0.845	0.726	0.746	70(31.3)	0.334	0.568	0.264
	GA	120(51.5)	192(49.1)				98(43.8)			
	AA	44(18.9)	78(19.9)				56(25.0)			
rs4833837	AA	189(78.1)	325(80.4)	0.61	0.474	0.751	185(81.1)	0.67	0.667	0.401
	GA	50(20.7)	72(17.8)				42(18.4)			
	GG	3(1.2)	7(1.7)				1(0.4)			
rs2221903	AA	176(73.6)	324(80.2)	0.063	0.053	0.061	185(81.1)	0.07	0.132	0.021 (11.72,1.46-94.13)
	GA	53(22.2)	73(18.1)				42(18.4)			
	GG	10(4.2)	7(1.7)				1(0.4)			
rs2055979	GG	92(38.5)	143(36.4)	0.808	0.595	0.857	91(41.6)	0.731	0.455	0.572
	GT	108(45.2)	188(47.8)				96(43.8)			
	TT	39(16.3)	62(15.8)				32(14.6)			
IL-21R										
rs3093301	CC	64(26.4)	111(27.5)	0.571	0.762	0.391	81(35.7)	0.079	0.023 (1.61, 1.07-2.42)	0.896
	TC	128(52.9)	197(48.9)				100(44.1)			
	TT	50(20.7)	95(23.6)				46(20.3)			
rs2285452	GG	185(77.7)	318(78.7)	0.823	0.707	0.999	193(85.0)	0.051	0.031 (1.71, 1.05-2.80)	0.435
	GA	52(21.8)	83(20.5)				32(14.1)			
	AA	1(0.4)	3(0.7)				2(0.9)			

### Association of the IL-21R gene polymorphisms with GD and HT

As shown in Table 1, the rs3093301 and rs2285452 SNPs in IL-21R showed no association with GD or HT (P = 0.797-0.068).

Nominal associations were found between rs3093301 and rs2285452 and HT when the dominant genetic model (CC *vs*. TC + TT, P = 0.023, OR = 1.61 95% CI = 1.070-2.42); GG *vs*. GT + TT, P = 0.031, OR = 1.71 95%CI = 1.05-2.80, respectively) was used for analysis. Genotype analysis of rs3093301 and rs2285452 using additive and recessive genetic model did not indicate any significant difference between HT patients and controls (Table 3). For rs3093301 and rs2285452, no significant different distribution was found in GD patients and controls when different genetic models was used (showed by Table 3).

### Haplotype analysis in GD and HT

As shown in Table 4, rs4833837 and rs2221903 (r-square = 0.901) were identified as an LD block using the Haploview 4.1. The total frequency of haplotypes listed in Table 5 was more than 99.0% in every group. AA containing major alleles of these two SNPs was associated with increased susceptibility to GD with an OR of 1.50 (95% CI =1.08-2.09, P = 0.016), and to HT with an OR of 1.69 (95% CI =1.14-2.52, P = 0.009) (Table 5).

**Table 4:** The linkage disequilibrium analysis of the SNPs in IL-21 and IL-21R gene

**r-square**			
IL-21	rs4833837	rs2221903	rs2055979
rs907715	0.099	0.090	0.493
rs4833837	--	0.901	0.067
rs2221903	--	--	0.059
IL-21R	rs2285452		
rs3093301	0.369(0.018)		

**Table 5:** The frequencies of the haplotypes of IL-21 gene in patients and controls

**Reference SNP**		**Frequency %**			**GD**		**HT**	
**rs4833837**	**rs2221903**	**Control**	**GD**	**HT**	**p**	**OR (95% CI)**	**p**	**OR (95% CI)**
A	A	84.7	89.3	90.4	0.016	1.500 (1.08-2.09)	0.009	1.694 (1.14-2.52)
G	G	11.6	10.7	9.6	0.640	0.92 (0.64-1.31)	0.340	0.816 (0.54-1.24)
A	G	3.7	0.0	0.0	4.0E-8	--	4.0E-4	--

## Discussion

IL-21 is a member of the common-gamma chain family of cytokines with immunoregulatory activity. IL-21R has been shown to form a heterodimeric receptor complex with the common gamma-chain. IL-21 binding with this receptor leads to the activation of multiple downstream signaling molecules, including JAK1, JAK3, STAT1 and STAT3, therefore affects the innate and adaptive immune responses by inducing the differentiation, proliferation and activity of multiple target cells. The dysregulation of IL-21 and IL-21R plays a role in multiple immune-mediated diseases, including SLE [3], psoriasis [5], RA [4] and other chronic inflammatory diseases [18]. Like other autoimmune diseases, GD and HT are chronic diseases initiated by the loss of immunological tolerance to self-antigens. Previous studies indicate that some immune-related genes may participate in the development of AITDs.

In this study, we found a significant association between GD or HT and rs2221903 located in the IL-21 gene region, and the frequencies of the haplotypes in the IL-21 gene that consists of rs4833837 and rs2221903 in GD and HT patients were significantly different from controls. For rs3093301 and rs2285452 polymorphisms of the IL-21R gene, there were significantly different distributions of these genotypes between HT patients and the controls.

In different from Jia’s study [6], we did not find the rs907715 SNP linkage with GD. In Jia’s report, the AA genotype frequency was lower in GD patients (19.4%) than in controls (30%). The gene frequency in patients of our study was similar to that of Jia’s study, but different in the two control groups. In our study the frequency of AA genotype in the control group was 18.9%, which is similar to the frequency in the HapMap-CHB population. This may be the geographical or selection variation that results in this difference. It should be noted that the lack of association in our study does not completely exclude the possibility of IL-21R as a candidate gene for GD because of the following three reasons: 1) The average MAF of the six SNPs is 0.271, which gives our study genetic power of about 0.8 for GD and 0.7 for HT group with an OR of homozygote 2.0, and of heterozygote 1.5, therefore studies with a larger sample size are necessary to confirm whether patients with these SNPs have more risk for development of GD. 2) As many other immunologic disorders, AITDs is believed to derive from a multiple network of various susceptible loci, which exert synergic or additive effects, but each locus may play a small role [19]. 3) 13 tag-SNPs in the HapMap-CHB population and 17 in the HapMap-CEU population covered a 49.8 kb on 16p11, where IL-21R gene located. And we just explored the association between the two tag-SNPs of them and AITDs in Chinese cohort.

In genetics, a recessive gene is an allele that causes a phenotype (visible or detectable characteristic) that is only seen in a homozygous genotype (an organism that has two copies of the same allele) and never in a heterozygous genotype. While dominance is a relationship between alleles of a gene, in which one allele masks the expression (phenotype) of another allele at the same locus. Our study didn’t find any synergistic effect of the risk genotype. And we were unable to conclude whether these two genes were either recessive or dominant over the other gene.

IL-21 is located on chromosome 4q26-27, which is close to IL-2, a region that has been linked to AITDs susceptibility [17]. Jia’s et al. found rs907715 of IL-21 gene being associated with GD in a Chinese population while Plagnol found only rs2069763 and did not find association of rs2069762 and rs6822844 of IL-2 with GD, maybe there is ethnicity or regional effect contributing to these results differences. Therefore, we can not exclude that the SNPs of the IL-21 and IL-21R genes are only an indicator of a candidate gene contributing to AITDs. Further studies such as screening the rest common variations in IL21R gene, identifying the causal SNP for this association, and exploring gene-environment interaction in GD and HT cohort studies are required to clarify the effect of IL-21 and IL-21R on AITDs susceptibility. Replication of these associations between IL-21 and IL-21R gene and AITDs is also required in larger independent databases of different cohorts.

## Conclusion

In conclusion, our study confirmed the synergic effect of the IL-21 SNPs in the development of GD. In addition, to the best of our knowledge, we are the first to report the association of the IL-21 and IL-21R SNPs with an increased risk of HT.

## Competing interests

The authors report no competing interests. The authors alone are responsible for the content and writing of the paper.

## Authors’ contributions

WXX and YFZ recruited the subjects and participated in the sequence alignment. LX and WJJ extracted genomic DNA from peripheral blood leukocytes. XHS carried out the molecular genetic studies, participated in the sequence alignment. JZ carried out the immunoassays and drafted the manuscript. SFM participated in the design of the study and performed the statistical analysis. JAZ and LHZ conceived of the study, and participated in its design and coordination. All authors read and approved the final manuscript.

## Pre-publication history

The pre-publication history for this paper can be accessed here:

http://www.biomedcentral.com/1472-6823/13/26/prepub

## References

[B1] Ozaki K Kikly K Michalovich D Young PR Leonard WJ Cloning of a type I cytokine receptor most related to the IL-2 receptor beta chain Proc Natl Acad Sci USA 2000 97 1439 1444 10.1073/pnas.200360997 PMC17218 11016959

[B2] Parrish-Novak J Dillon SR Nelson A Hammond A Sprecher C Gross JA Johnston J Madden K Xu W West J Schrader S Burkhead S Heipel M Brandt C Kuijper JL Kramer J Conklin D Presnell SR Berry J Shiota F Bort S Hambly K Mudri S Clegg C Moore M Grant FJ Lofton-Day C Gilbert T Rayond F Ching A Interleukin 21 and its receptor are involved in NK cell expansion and regulation of lymphocyte function Nature 2000 408 57 63 10.1038/35040504 11081504

[B3] Bubier JA Sproule TJ Foreman O Spolski R Shaffer DJ Morse HC 3rd Leonard WJ Roopenian DC A critical role for IL-21 receptor signaling in the pathogenesis of systemic lupus erythematosus in BXSB-Yaa mice Proc Natl Acad Sci USA 2009 106 1518 1523 10.1073/pnas.0807309106 19164519 PMC2635818

[B4] Li J Shen W Kong K Liu Z Interleukin −21 induces T-cell activation and proinflammatory cytokine secretion in rheumatoid arthritis Scand J Immunol 2006 64 515 522 10.1111/j.1365-3083.2006.01795.x 17032244

[B5] Yuan SL Jiang L Zhang XL Li SF Duan HM Wang XF Serum IL-21 level in patients with primary Sjogren’s syndrome and clinical significance of IL-21 Xi Bao Yu Fen Zi Mian Yi Xue Za Zhi 2007 23 124 126 17286904

[B6] Jia HY Zhang ZG Gu XJ Guo T Cui B Ning G Zhao YJ Association between interleukin 21 and Graves’ disease Genet Mol Res 2011 10 3338 3346 10.4238/2011.October.31.6 22057994

[B7] Wellcome Trust Case Control Consortium Genome-wide association study of 14,000 cases of seven common diseases and 3,000 shared controls Nature 2007 447 661 678 10.1038/nature05911 17554300 PMC2719288

[B8] Zhernakova A Alizadeh BZ Bevova M Van Leeuwen MA Coenen MJ Franke B Franke L Posthumus MD Van Heel DA Van der Steege G Radstake TR Barrera P Roep BO Koeleman BP Wijmenga C Novel association in chromosome 4q27 region with rheumatoid arthritis and confirmation of type 1 diabetes point to a general risk locus for autoimmune diseases Am J Hum Genet 2007 81 1284 1288 10.1086/522037 17999365 PMC2276343

[B9] Hinks A Eyre S Ke X Barton A Martin P Flynn E Packham J Worthington J Thomson W Association of the AFF3 gene and IL2/IL21 gene region with juvenile idiopathic arthritis Genes Immun 2010 11 194 198 10.1038/gene.2009.105 20072139 PMC2845517

[B10] Liu Y Helms C Liao W Zaba LC Duan S Gardner J Wise C Miner A Malloy MJ Pullinger CR Kane JP Saccone S Worthington J Bruce I Kwok PY Menter A Krueger J Barton A Saccone NL Bowcock AM A genome-wide association study of psoriasis and psoriatic arthritis identifies new disease loci PLoS Genet 2008 4 e1000041 10.1371/journal.pgen.1000041 18369459 PMC2274885

[B11] Adamovic S Amundsen SS Lie BA Gudjónsdóttir AH Ascher H Ek J Van Heel DA Nilsson S Sollid LM Torinsson Naluai A Association study of IL2/IL21 and FcgRIIa: significant association with the IL2/IL21 region in Scandinavian coeliac disease families Genes Immun 2008 9 364 367 10.1038/gene.2008.27 18418394

[B12] Garner CP Murray JA Ding YC Tien Z Van Heel DA Neuhausen SL Replication of celiac disease UK genome-wide association study results in a US population Hum Mol Genet 2009 18 4219 4225 10.1093/hmg/ddp364 19648293 PMC2758145

[B13] Festen EA Goyette P Scott R Annese V Zhernakova A Lian J Lefèbvre C Brant SR Cho JH Silverberg MS Taylor KD De Jong DJ Stokkers PC Mcgovern D Palmieri O Achkar JP Xavier RJ Daly MJ Duerr RH Wijmenga C Weersma RK Rioux JD Genetic variants in the region harbouring IL2/IL21 associated with ulcerative colitis Gut 2009 5 799 804 19201773 10.1136/gut.2008.166918 PMC2757103

[B14] Fedetz M Ndagire D Fernandez O Leyva L Guerrero M Arnal C Lucas M Izquierdo G Delgado C Alcina A Matesanz F Multiple sclerosis association study with the TENR-IL2-IL21 region in a Spanish population Tissue Antigens 2009 74 244 247 10.1111/j.1399-0039.2009.01298.x 19523143

[B15] Sawalha AH Kaufman KM Kelly JA Adler AJ Aberle T Kilpatrick J Wakeland EK Li QZ Wandstrat AE Karp DR James JA Merrill JT Lipsky P Harley JB Genetic association of interleukin-21 polymorphisms with systemic lupus erythematosus Ann Rheum Dis 2008 67 458 461 17720724 10.1136/ard.2007.075424

[B16] Webb R Merrill JT Kelly JA Sestak A Kaufman KM Langefeld CD Ziegler J Kimberly RP Edberg JC Ramsey-Goldman R Petri M Reveille JD Alarcón GS Vilá LM Alarcón-Riquelme ME James JA Gilkeson GS Jacob CO Moser KL Gaffney PM Vyse TJ Nath SK Lipsky P Harley JB Sawalha AH A polymorphism within IL21R confers risk for systemic lupus erythematosus Arthritis Rheum 2009 60 2402 2407 10.1002/art.24658 19644854 PMC2782592

[B17] Plagnol V Howson JM Smyth DJ Walker N Hafler JP Wallace C Stevens H Jackson L Simmonds MJ Bingley PJ Gough SC Todd JA Type 1 Diabetes Genetics Consortium Genome-wide association analysis of autoantibody positivity in type 1 diabetes cases PLoS Genet 2011 7 e1002216 10.1371/journal.pgen.1002216 21829393 PMC3150451

[B18] De Nitto D Sarra M Pallone F Monteleone G Interleukin-21 triggers effector cell responses in the gut World J Gastroenterol 2010 16 3638 3641 10.3748/wjg.v16.i29.3638 20677335 PMC2915423

[B19] Leonard WJ Spolski R Interleukin-21: a modulator of lymphoid proliferation, apoptosis and differentiation Nat Rev Immunol 2005 5 688 698 10.1038/nri1688 16138102

